# Heat Stress Modulates Brain Monoamines and Their Metabolites Production in Broiler Chickens Co-Infected with *Clostridium perfringens* Type A and *Eimeria* spp.

**DOI:** 10.3390/vetsci6010004

**Published:** 2019-01-09

**Authors:** Atilio Sersun Calefi, Juliana Garcia da Silva Fonseca, Catarina Augusta de Queiroz Nunes, Ana Paula Nascimento Lima, Wanderley Moreno Quinteiro-Filho, Jorge Camilo Flório, Adriano Zager, Antonio José Piantino Ferreira, João Palermo-Neto

**Affiliations:** Department of Pathology, School of Veterinary Medicine and Animal Science, University of São Paulo, Av. Prof. Dr. Orlando Marques de Paiva, 87, São Paulo 05508-900, Brazil; julianagdsf@gmail.com (J.G.d.S.F.); catarina.queirooz@gmail.com (C.A.d.Q.N.); paulanlima@gmail.com (A.P.N.L.); quinteirofilho@gmail.com (W.M.Q.-F.); jflorio@usp.br (J.C.F.); adrianozager@gmail.com (A.Z.); ajpferr@usp.br (A.J.P.F.); jpalermo@usp.br (J.P.-N.)

**Keywords:** neuroimmunomodulation, sickness behavior, avian necrotic enteritis, gut-brain axis, hypothalamus-pituitary-adrenal axis

## Abstract

Heat stress has been related to the impairment of behavioral and immunological parameters in broiler chickens. However, the literature is not clear on the involvement of neuroimmune interactions in a heat stress situation associated with bacterial and parasitic infections. The present study evaluated the production of monoamines and their metabolites in brain regions (rostral pallium, hypothalamus, brain stem, and midbrain) in broiler chickens submitted to chronic heat stress and/or infection and co-infection with *Eimeria* spp. and *Clostridium perfringens* type A. The heat stress and avian necrotic enteritis (NE) modulated the neurochemical profile of monoamines in different areas of the central nervous system, in particular, those related to the activity of the hypothalamus-hypophysis-adrenal (HPA) axis that is responsible for sickness behavior. *C. perfringens* and/or *Eimeria* infection, heat stress increased 5-hydroxytryptamine (5-HT), 4,4 dihydroxyphenylacetic acid (DOPAC), and DOPAC/dopamine (DA) in the rostral pallium; 3-methoxy-4-hydroxyphenylethylene glycol (MHPG), homovanillic acid (HVA), HVA/DA, DOPAC/DA, and 5-hydroxyindoleacetic acid (5-HIAA)/5-HT in the hypothalamus; MHPG, 5-HIAA/5-HT, DOPAC/DA, and HVA/DA in the midbrain; and MHPG, DOPAC, HVA, HVA/DA, DOPAC/DA, and 5-HIAA/5-HT in the brainstem. Heat stress decreased noradrenaline + norepinephrine (NOR + AD) in all brain regions analyzed; 5-HT in the hypothalamus, midbrain, and brainstem; and DA in the midbrain. The results also showed the existence and activity of the brain-gut axis in broiler chickens. The brain neurochemical profile and corticosterone production are consistent with those observed in chronic stressed mammals, in animals with sickness behavior, and an overloading of the HPA axis.

## 1. Introduction

Besedovsky et al. in 1977 [1] showed, for the first time, that immune challenge with sheep erythrocytes activated hypothalamic neurons in rats. The authors suggested that immune system activation increases norepinephrine (NOR) turnover in the rat hypothalamus. These studies marked the beginning of research related to the effects of the immune system on nervous system activity [2].

Currently, it is known that cytokines released by leukocytes activate neurons in the brain [3]. The major pathways proposed to explain the mechanism by which cytokines activate different central nervous system (CNS) regions are not fully understood. It is suggested that the mechanisms of cytokine activation of the CNS are related to the following: (i) The penetration of cytokines into the CNS through the openings in the blood-brain barrier (BBB) (in circumventricular organs); (ii) the passing of cytokines through the BBB via local membrane transporters; (iii) neuronal activation produced by the stimulation of the afferent peripheral nervous system, such as the vagus nerve; (iv) by activated cells, e.g., endothelial cells, which produce and release molecules that cross the BBB; and finally, (v) the activation of the leukocytes that produce and release cytokines directly into the CNS (for more details, see the review of [4]).

NOR and 5-hydroxytryptamine (5-HT) participate in the activation of the hypothalamus- hypophysis-adrenal axis (HPA) axis and the hypophysary release of adrenocorticotropic hormone (ACTH); these amines, in turn, have been associated with peripheral neuroimmune activation, i.e., being triggered by the release of IL-1, IL-6, and TNF-α [5]. In this context, it is known that activation of the HPA axis produces increased cortisol secretion and sickness behavior in humans [6], as well as in other animals [6,7]. The expression of sickness behavior is associated with a decrease in feed intake and increase in sleepiness with consequent weight loss in poultry [8,9].

The modulation of the immune system by the CNS is also important in the manifestation of stress effects [10]. Physical (environmental temperature) and psychological (social disruption) stress are known to activate the HPA axis [11,12]. Physical stress increases the concentration and turnover of hypothalamic 5-HT; however, the chronicity and frequency of stress have been linked to decreased levels of 5-HT and NOR and increased 5-hydroxyindoleacetic acid (5-HIAA) concentration in the hypothalamus, pons, and medulla oblongata [13,14,15].

A recent study from our laboratory [16] has shown that heat stress and intestinal inflammation increase the neuronal activation in hypothalamic nuclei (paraventricular nucleus [PVN] and medial preoptic nucleus [POM]) and decrease neuronal activation in the amygdala, a component of the limbic system, in an avian model. Some studies conducted with chronic stress in mammals indicate that activation of the HPA axis is related to an increased activity of PVN and POM neurons as well as to a breakdown in the connection of the limbic system to the prefrontal cortex [17,18,19]. The corresponding brain structure to the prefrontal cortex in birds is called nidopallium [20]. These findings clearly show the existence of a modulation of the CNS related to the immune system.

Thus, the aim of the current study is to analyze the changes in the neurochemical profile in the rostral pallium, hypothalamus, brain stem, and midbrain of broiler chickens after activation of the brain-gut axis by chronic heat stress and/or by the infection and co-infection with *Eimeria* spp. and *Clostridium perfringens* type A.

## 2. Materials and Methods

### 2.1. Animals

One-day-old broilers were housed in controlled environment on the premises of the School of Veterinary Medicine and Animal Science, University of São Paulo, Brazil. Eighty male broilers (Cobb 500) were maintained in isolation chambers (Alesco, São Paulo, Brazil) containing high-efficiency particulate air filters (HEPA) from the first day of life (ED1) to the last experimental day (ED23). The birds received water *ad libitum*. The relative humidity was monitored and controlled (not less than 45%). Animals were used and maintained with the approval of the Ethics Committee on Animal Use from the School of Veterinary Medicine and Animal Science (CEUA/FMVZ), Brazil (permit number 3071/2013).

### 2.2. Group Formation

On ED1, broilers were randomly allocated into eight groups of 10 animals in each group as follows: A control group (C), a group of animals infected with *Eimeria* spp. (Ei), a group of animals infected with *C. perfringens* (Cp), a group of animals co-infected with *C. perfringens* and *Eimeria* spp. (Cp + Ei), a control group stressed by heat (C/HS34), a group of animals infected by *Eimeria* spp. and stressed by heat (Ei/HS34), a group of animals infected with *C. perfringens* and stressed by heat (Cp/HS34), and a group of animals co-infected with *C. perfringens* and *Eimeria* spp. and stressed by heat (Cp + Ei/HS34). The groups were allocated in duplicate containing five animals each. The individual chickens were used as the experimental unit and duplicate was used as a blocking factor.

### 2.3. Heat Stress Protocol

The broilers of the heat stressed groups (C/HS34, Ei/HS34, Cp/HS34, and Cp + Ei/HS34) were kept at a room temperature of 34±1 °C from ED17 to ED23. During this period, the animals that were not stressed by heat (C, Cp, Ei, and Cp + Ei) were maintained at ambient temperatures compatible with those recommended for the lineage with ±1 °C of ambient variation.

### 2.4. Eimeria spp. Infection Protocol

On ED18, animals received attenuated live vaccine BioCoccivet-R (Biovet, SP, Brazil) by gavage at a concentration 30 times higher than recommended by the manufacturer (groups Ei, Cp + Ei, Ei/HS34, and Cp + Ei/HS34). This vaccine contains live oocysts from precocious strains of *Eimeria acervulina*, *E. brunetti*, *E. hagani*, *E. tenella*, *E. necatrix*, *E. mivati*, *E. maxima*, and *E. praecox*.

### 2.5. Inoculum Preparation and C. perfringens Infection Protocol

The current experimental protocol was previously established by Calefi et al. [21]. A pathogenic strain of *C. perfringens* type A (strain CP8.2, genotype for productions of Tpel and α toxins, NetB negative) has been maintained in our laboratory in glycerol at −80 °C. Briefly, the inoculum was prepared by two alternate cultures in thioglycolate broth medium (Becton, Dickinson and Company, Franklin Lakes, NJ, USA) with 2% yeast extract (Becton, Dickinson and Company, Franklin Lakes, NJ, USA) and cooked meat medium (Becton, Dickinson and Company, Franklin Lakes, NJ, USA). Bacteria were given to broilers from ED15 to ED19 in a final concentration of approximately 1 × 10^4^ cfu/g of feed. From ED1 to ED23, animals received feed with 24% crude protein without antimicrobial agent. Starting at ED19, the groups (Cp, Cp + Ei, Cp/HS34, and Cp + Ei/HS34) received the culture medium containing a pathogenic strain of *C. perfringens* type A mixed with the animal feed at a ratio of 1:1 (*v*/*v*). The control groups (C and C/HS34) received only feed without any pathogen mixed with brain heart infusion culture medium (BHI). The infection occurred through voluntary feed intake containing the BHI with *C. perfringens*. The volume of the inoculum was standardized to be completely consumed within a maximum period of 6 h.

### 2.6. Histopathological Evaluation

On ED23, 2 cm long sections of the duodenum (portion before the duodenal flexure), jejunum (the middle region between the end of the duodenum and Meckel’s diverticulum), and ileum (the middle region between Meckel’s diverticulum and the ileo-cecal junction) were collected and fixed in 10% formaldehyde for 48 h; subsequently, samples were embedded in paraffin following a standard procedure for tissue inclusion. Cross sections of 5 μm were stained with hematoxylin and eosin (HE) to perform the histopathological evaluation. To evaluate the morphology of bacteria adhered to necrosis foci Gram staining was performed.

For diagnosis of the lesions produced by the infection, the entire area of three transversal intestinal sections per small intestinal portion (duodenum, jejunum, and ileum) was evaluated for necrosis, inflammation (heterophils infiltration in villi and crypt and mononuclear infiltrate), fusion of villi, congestion, edema, and hemorrhage. Each lesion type was classified as without alterations (0), mild (score 1), moderate (score 2), and marked-to-severe (score 3) for necrosis, inflammation, congestion, edema, and hemorrhage, as suggested by Calefi et al. [21]. The fusion of villi was also scored as (1): Less than 3 fused villi per section, (2): Between 3 to 6 fused villi per section, and (3): More than 6 fused villi per section. A total score was determined by summing the scores obtained in all sections evaluated from the intestinal portions per animal.

### 2.7. Quantification of Serum Corticosterone

On ED23, blood samples were collected from the brachial veins of the animals of all groups, and the serum were obtained for hormonal quantification. The free corticosterone (corticosterone unbinded to corticosteroid binding globulin) levels were quantified by commercial ELISA kits (Arbor Assays Inc, Eisenhower, MI, USA) according to the manufacturer’s instructions, preceded by standardized dilutions. Serum corticosterone was determined with the aid of a standard curve expressed in nanogram of corticosterone per millilitre of serum (pg/mL); the results were multiplied by the dilution factor used in the test.

### 2.8. Collection and Processing of Brain Tissue

The animals were euthanized by decapitation. Immediately after, the brain was removed, and the rostral pallium (mainly composed by the nidopallium, also contain hyperpallium and mesopallium), midbrain, hypothalamus, and brainstem were quickly dissected out at 4 °C, frozen in liquid nitrogen, and stored at −80 °C until use. Brain tissue was homogenized by sonication in 0.1 M perchloric acid solution containing 0.02% of sodium metabisulfite (Na_2_S_2_O_5_) and a known concentration of 3,4-dihydroxybenzylamine (DHBA), which was used as an internal standard. The homogenate was centrifuged at 10,000× *g* for 15 min, and the supernatant was removed for the measurement of neurotransmitters.

### 2.9. Sample Preparation and Neurotransmitter Quantification

High performance liquid chromatography (HPLC) was used coupled to electrochemical detection (HPLC-ED; Shimadzu Model 20A, Kyoto, Japan) for neurochemical measurements. HPLC-ED consists of an autosampler with a variable-volume injection valve with 1 to 100 μL quaternary flow pumps, a system controller, a chromatographic column measuring 150 × 4.6 mm in diameter (Shimpak—ODS C18) with a strip and an electrochemical detector, AntecDecade. The technique used was chromatography on reverse phase with ion pairing in the mobile phase at 50 °C. A working solution consisted of 0.02 M citrate buffer, methanol 92/8 (*v*/*v*) ethylenediamine tetraacetic acid (EDTA), and 0.12 nM 0.0556% 1-heptanesulfonic acid (HSA). The pH was adjusted to 2.64 by the use of orthophosphoric acid (H_3_PO_4_). The electrode detector was maintained at 0.8 V. The standard solutions used were 1 nM concentrations of 4,4 dihydroxyphenylacetic acid (DOPAC), homovanillic acid (HVA), 5-HIAA, dopamine (DA), serotonin (5-HT), NOR, epinephrine (AD) vanillylmandelic acid (VMA), and 3-methoxy-4-hydroxyphenylglycol (MHPG). The standards were injected at the beginning and end of each determination, and the variation coefficients were always less than 10%. The ratios of DOPAC/DA, HVA/DA, and 5-HIAA/5-HT were calculated as indicators of DA and 5-HT turnover.

### 2.10. Statistical Analysis

Heat maps and cluster analysis were used for the analysis in averages variations of neurotransmitters in the brain structures from the animals of the different experimental groups with the aid of the function *heatmap.2* from the *gplots* package (version 2.12.1, Boston, MA, USA) using the statistical software R (version 3.2.3; R foundation for statistical computing, Vienna, Austria). The average neurotransmitter concentrations of the experimental groups were centered in relation to the control group. To assess the similarity of the monoamines and metabolites production between two groups a score was computed and the data were centered to normalize the values. The heat maps were reorganized in their rows and columns to identify similarities between neurotransmitters and were based on the Euclidean distance and complete agglomeration method, i.e., rows and columns were sorted based on the hierarchical clustering result. The colors were assigned to represent the amine quantitation values in each brain portion. A color bar which represents the relation between colors and values were generated at the top left side of the figures, and a histogram shows the overall frequency of the data in each score. The distances between neurotransmitters by group are represented by dendrograms in the *x*- and *y*-axes of the graphic. This approach was preferred to replace conventional presentations to demonstrate similarities and dissimilarities of neurotransmitters between all analytes and experimental groups. Thus, subtle variations and clusters are easily depicted in one figure, a fact that is not evident in graphic presentations in bars and boxplots. Nonparametric data were analyzed by Kruskal-Wallis test followed by multiple comparison using the function *kruskalmc* from the package *pgirmess* (version 1.6.5, Besançon, France) using the statistical software R (version 3.2.3, Vienna, Austria). The differences were considered significant at *p* ≤ 0.05.

## 3. Results

### 3.1. Histopathological Evaluation

**Figure 1:**
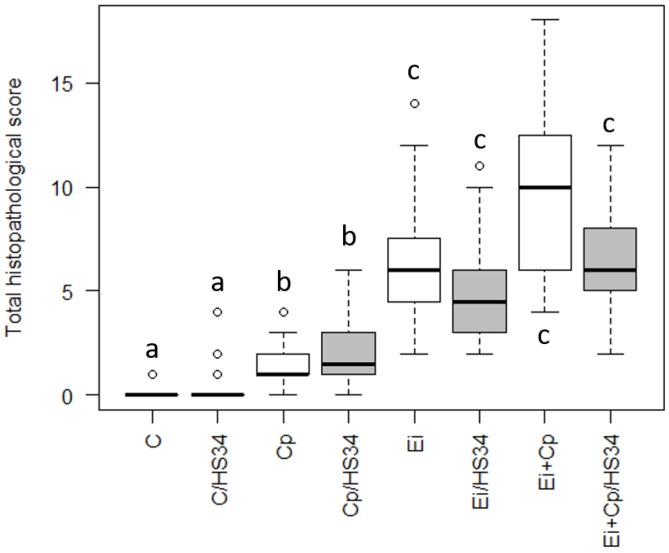
Scores of histopathological lesions in small intestine of broilers. The boxplots represent the median plus the maximum and minimum scores by group. The different letters assigned to each boxplot indicate significant differences for *p* ≤ 0.05 (Krukal-Wallis test). Control group (C); group infected with *Clostridium perfringens* (Cp); group infected with *Eimeria* spp. (Ei); group infected with *Clostridium perfringens* + *Eimeria* spp. (Ei + Cp). ‘/HS34’ beside the group name indicates the presence of heat stress.

The histopathological evaluation confirmed that the *C. perfringens* and *Eimeria* spp. infection were effective in causing tissue injury. The most serious lesions were observed in the co-infected animals, represented by severe diffuse necrotizing enteritis with adherent bacilli in the mucosa. The group Cp presented mild enteritis with focal to multifocal necrosis. In all infected animals with *Eimeria* spp. were observed stages of the coccidias in the tissue. The scores obtained were presented in Figure 1.

### 3.2. Quantification of Serum Corticosterone

**Figure 2:**
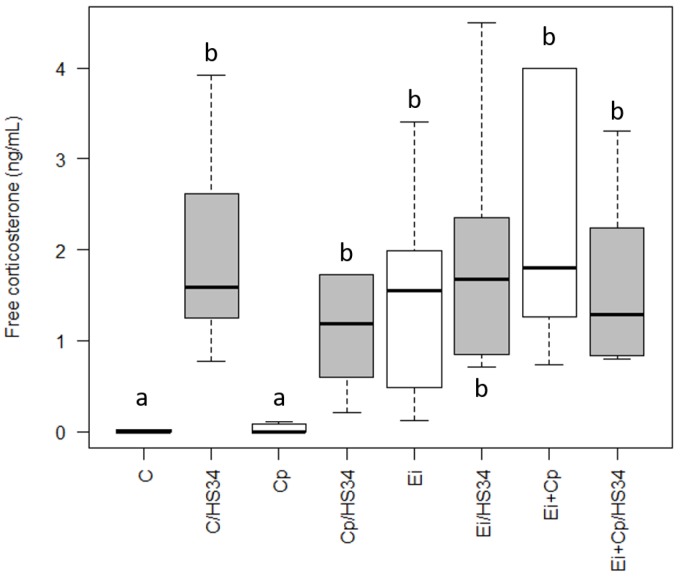
Quantification of serum levels of free corticosterone. The boxplots represent the median plus the maximum and minimum scores by group. The different letters assigned to each boxplot indicate significant differences *p* ≤ 0.05 (Krukal-Wallis test). Control group (C); group infected with *Eimeria* spp. (Ei); group infected with *Clostridium perfringens* (Cp); group infected with *Clostridium perfringens* + *Eimeria* spp. (Ei + Cp). ‘/HS34’ beside the group name indicates the presence of heat stress.

Heat stress increased the free corticosterone in the animals of the C/HS34 group compared to group C (*p* ≤ 0.05; Figure 2). The animals of groups C/HS34, Ei, Ei/HS34, Cp/HS34, Ei + Cp, and Ei + Cp/HS34 had higher levels of free corticosterone in serum when compared with that measured in control group animals and *C. perfringens* infected animals (Group C and Cp, respectively; *p* ≤ 0.05; Figure 2).

### 3.3. Neurotransmitter Quantification in Brain Structures

For better visualization of the overall changes in neurotransmitter levels by brain region, data are presented by structure analyzed (rostral pallium, hypothalamus, midbrain, and brainstem). The average and standard deviation from each brain monoamines and metabolites per group were analyzed with ANOVA and Tukey honest significant difference (TukeyHSD) as *post hoc* and are presented as Supplementary Tables.

#### 3.3.1. Rostral Pallium

**Figure 3:**
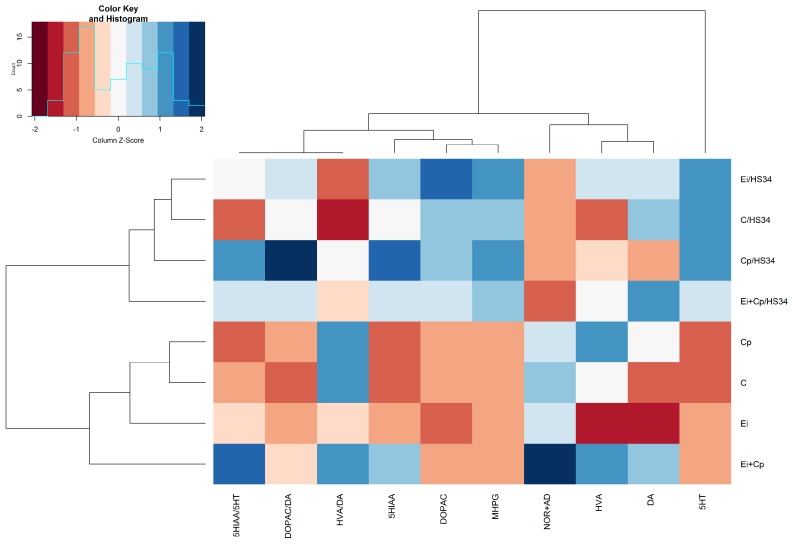
Heat map with the average concentrations of neurotransmitters in the rostral pallium. The data were normalized to the mean of the control group (C), followed by normalization for each neurotransmitter. Groupings in rows and columns were made to identify similarities between neurotransmitters and were based on the Euclidean distance and complete agglomeration method. The distances are represented by dendrograms in the *x*- and *y*-axes. Control group (C); group infected with *Eimeria* spp. (Ei); group infected with *Clostridium perfringens* (Cp); group infected with *Clostridium perfringens* +3 *Eimeria* spp. (Ei + Cp). ‘/HS34’ beside the group name indicates the presence of heat stress.

Figure 3 illustrates the stress-induced increase in levels of 5-HT (*p* ≤ 0.05 [C/HS34, Ei/HS34 and Cp/HS34 groups]), DOPAC (*p* ≤ 0.05 [C/HS34 and Cp/HS34 groups]), and the ratio of DOPAC/DA (*p* ≤ 0.05 [Cp/HS34 animals compared to those of the C group]) and decrease in mean levels of NOR + AD (*p* ≤ 0.05 [C/HS34, Cp/HS34, Ei/HS34, and Ei + Cp/HS34 groups]). Animals from the C/HS34, Ei/HS34, and Cp/HS34 groups exhibited an increase in 5-HIAA when compared to the respective unstressed groups (*p* ≤ 0.05); however, 5-HT turnover, as assessed by the 5-HIAA/5-HT ratio, was higher only in the Cp/HS34 and Ei/HS34 groups (*p* > 0.05). The brain monoamine quantification averages and standard errors by experimental group are shown in Supplementary Table S1.

#### 3.3.2. Hypothalamus

**Figure 4:**
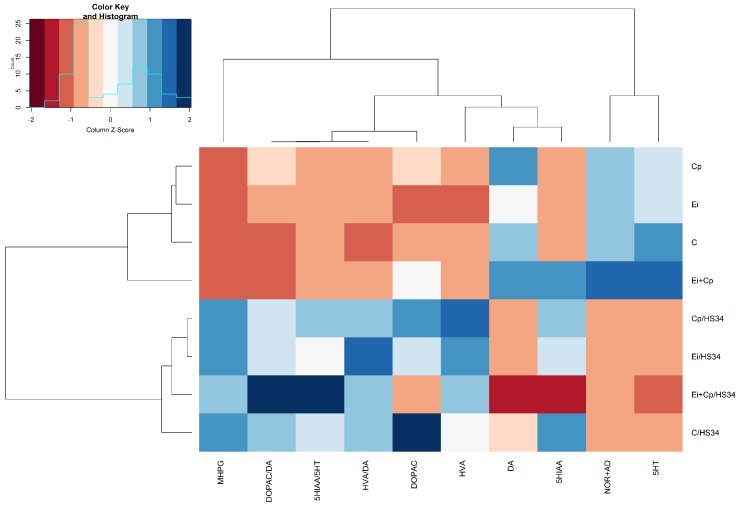
Heat map with the average concentrations of neurotransmitters in the hypothalamus. The data were normalized to the mean of the control group (C), followed by normalization for each neurotransmitter. Groupings in rows and columns were made to identify similarities between neurotransmitters and were based on the Euclidean distance and complete agglomeration method. The distances are represented by dendrograms in the *x*- and *y*-axes. Control group (C); group infected with *Eimeria* spp. (Ei); group infected with *Clostridium perfringens* (Cp); group infected with *Clostridium perfringens* + *Eimeria* spp. (Ei + Cp). ‘/HS34’ beside the group name indicates the presence of heat stress.

Figure 4 shows that heat stress increased the mean levels of MHPG (*p* ≤ 0.05 [C/HS34, Cp/HS34, Ei/HS34, and Ei + Cp/HS34 groups]), HVA (*p* ≤ 0.05 [C/HS34, Cp/HS34, Ei/HS34, and Ei + Cp/HS34 groups]), HVA/DA (*p* ≤ 0.05 [C/HS34, Cp/HS34, Ei/HS34, and Ei + Cp/HS34 groups]), DOPAC/DA (*p* ≤ 0.05 [Ei + Cp/HS34 group]), and 5-HIAA/5-HT (*p* ≤ 0.05 [Ei + Cp/HS34 group]) and decreased the mean levels of 5-HT (*p* ≤ 0.05 [C/HS34, Cp/HS34, Ei/HS34, and Ei + Cp/HS34 groups]) and NOR + AD (*p* ≤ 0.05 [C/HS34, Cp/HS34, Ei/HS34, and Ei + Cp/HS34 groups]) in animals of all stressed groups. Groups C/HS34, Ei/HS34, and Cp/HS34 demonstrated a reduction in DOPAC when compared to the respective unstressed groups (*p* > 0.05). The brain monoamine quantification averages and standard deviations by experimental group are shown in Supplementary Table S2.

#### 3.3.3. Midbrain

**Figure 5:**
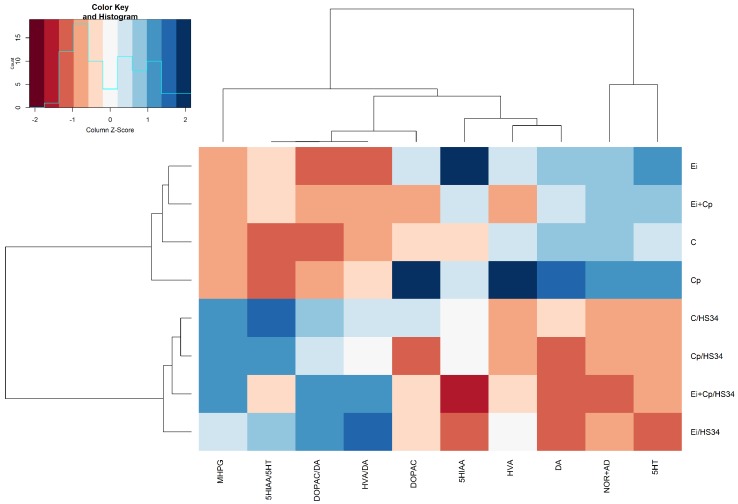
Heat map with the average concentrations of neurotransmitters in the midbrain. The data were normalized to the mean of the control group (C), followed by normalization for each neurotransmitter. Groupings in rows and columns were made to identify similarities between neurotransmitters and were based on the Euclidean distance and complete agglomeration method. The distances are represented by dendrograms in the x- and y-axes. Control group (C); group infected with *Eimeria* spp. (Ei); group infected with *Clostridium perfringens* (Cp); group infected with *Clostridium perfringens* + *Eimeria* spp. (Ei + Cp). ‘/HS34’ beside the group name indicates the presence of heat stress.

Figure 5 illustrates that heat stress increased the mean levels of MHPG (*p* ≤ 0.05 [C/HS34, Cp/HS34, Ei/HS34, and Ei + Cp/HS34 groups]), 5-HIAA/5-HT (*p* ≤ 0.05 [C/HS34 group]), DOPAC/DA (*p* ≤ 0.05 [Ei/HS34 group]), and HVA/DA (*p* > 0.05) and decreased the mean levels of DA (*p* ≤ 0.05 [C/HS34, Cp/HS34, Ei/HS34, and Ei + Cp/HS34 groups]), NOR + AD (*p* ≤ 0.05 [C/HS34, Cp/HS34, Ei/HS34, and Ei + Cp/HS34 groups]), and 5-HT (*p* ≤ 0.05 [Cp/HS34, Ei/HS34, and Ei + Cp/HS34 groups]). The animals of the C/HS34, Ei/HS34, and Cp/HS34 groups exhibited a decrease in HVA when compared to the respective unstressed groups (*p* > 0.05). The brain monoamine quantitation averages and standard errors by experimental group are shown in Supplementary Table S3.

#### 3.3.4. Brainstem

**Figure 6:**
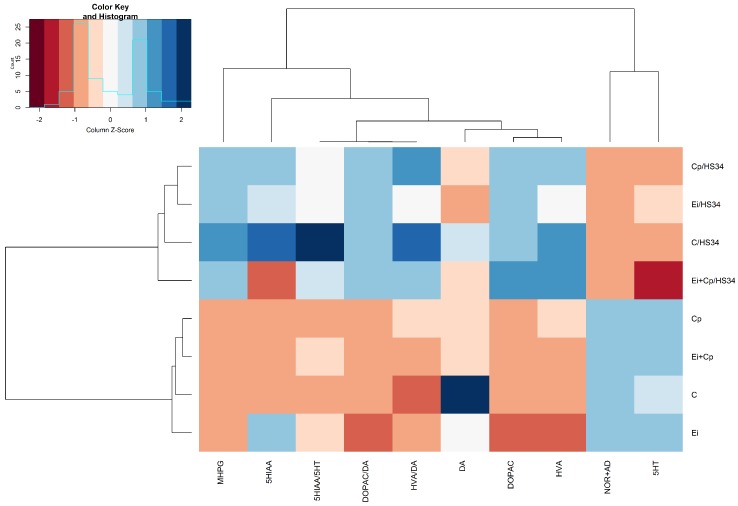
Heat map with the average concentrations of neurotransmitters in the brainstem. The data were normalized to the mean of the control group (C), followed by normalization for each neurotransmitter. Groupings in rows and columns were made to identify similarities between neurotransmitters and were based on the Euclidean distance and complete agglomeration method. The distances are represented by dendrograms in the x- and y-axes. Control group (C); group infected with *Eimeria* spp. (Ei); group infected with *Clostridium perfringens* (Cp); group infected with *Clostridium perfringens* + *Eimeria* spp. (Ei + Cp). ‘/HS34’ beside the group name indicates the presence of heat stress.

Figure 6 shows that heat stress increased the mean levels of MHPG (*p* ≤ 0.05 [C/HS34, Cp/HS34, Ei/HS34, and Ei + Cp/HS34 groups]), DOPAC (*p* ≤ 0.05 [C/HS34, Ei/HS34, and Ei + Cp/HS34 groups]), HVA (*p* > 0.05), HVA/DA (*p* > 0.05), DOPAC/DA (*p* ≤ 0.05 [Cp/HS34, Ei/HS34, and Ei + Cp/HS34 groups]), and 5-HIAA/5-HT (*p* ≤ 0.05 [C/HS34 group]) and decreased the mean levels of NOR + AD (*p* ≤ 0.05 [C/HS34, Cp/HS34, Ei/HS34, and Ei + Cp/HS34 groups]) and 5-HT (*p* ≤ 0.05 [C/HS34, Cp/HS34, Ei/HS34, and Ei + Cp/HS34 groups]). The animals of the C/HS34, Ei/HS34, and Cp/HS34 groups showed an increase in the 5-HIAA/5-HT ratio when compared to the respective unstressed groups (*p* > 0.05). The brain monoamine quantitation averages and standard errors by experimental group are shown in Supplementary Table S4.

## 4. Discussion

This experimental model of necrotic enteritis by *Eimeria* spp. and *C. perfringens* co-infection was used in this study in an attempt to reproduce the reality observed in the field [22]: Indeed, coccidiosis has been considered the most common occurrence observed in the course of clinical necrotic enteritis (NE) [23,24]. According to Parish [23], the association of *Eimeria* spp. with *C. perfringens* induces bowel necrosis with massive recruitment of heterophils to the site of infection.

This injury, in turn, has the potential to induce cytokine release from the inflamed site into circulation, activating the CNS and modulating animal behavior, as already suggested in other experimental contexts [4,25]. This experimental model sought thereby, to simulate the complex interactions between the intestinal infectious processes to analyze the neurochemical data from broilers in the presence or absence of heat stress.

Previous studies have shown that intestinal infection with C. perfringens *per se* increases the production of cytokines in the intestine and modulates leukocyte populations in the spleen and in systemic circulation [26,27]. Thus, the cytokines released in the bird gut might have entered the CNS, activating the HPA axis, which in turn, would have modulated the behavior of the animals, as previously suggested [5,16,28]. In mammals, activation of the HPA axis by LPS injection induces a neurochemical response characterized by increased levels of NOR, DA, and 5-HT [4,29]. IL-1, in turn, increases mainly NOR and 5-HT; TNF, however, induces increases in NOR and 5-HT only when present in high concentrations [3,30]. Indeed, our study demonstrated that infection with *C. perfringens* (Cp group) increased the levels of DA in the rostral pallium, hypothalamus and midbrain. The co-infection with *C. perfringens* and *Eimeria* spp. increased the levels of DA only in the rostral pallium and hypothalamus. When infection with *Eimeria* spp. reduce the concentrations of DA and the HVA/DA ratio in the brain structures analyzed, that is, the infection with *C. perfringens* is related to the activation of dopaminergic pathways. A possible explanation for these findings may be in the release of IL-1 by the infectious process [29].

The severity of the infection process also seems to be related to the brain monoamines analyzed. The increase in NOR + AD was only found in the Ei + Cp group and was not observed in animals of the Ei and Cp groups. It is known that co-infection produces more tissue damage than isolated infection with pathogens [22]. The animals of the Ei + Cp group potentially had a greater activation of the HPA axis due to increased harmful processes and intestinal inflammation, which would result in an increase in the NOR + AD concentrations in the CNS of the broilers [31]. In addition, co-infection increased 5-HT and 5-HIAA concentrations in the rostral pallium, hypothalamus, and brainstem, findings not observed with isolated infections. However, we noted that any type of intestinal infection activated the serotoninergic system of the midbrain, represented by increased levels of 5-HT and 5-HIAA. Thus, the activation of serotonergic pathways in the rostral pallium, hypothalamus, and midbrain seems proportional to the magnitude of the infection and intestinal damage; in this context, the serotonergic system in the midbrain region would be more sensitive to the intestinal infectious process. There is also a possibility that the activation of the midbrain serotonergic system modulates the dopaminergic system, contributing to changes in DA and HVA levels observed in the animals with infectious processes [32,33]. In addition, we can hypothesize that cytokine production in CNS is related to a kynurenine pathway activation, which deprives the 5-HT pathway of tryptophan, and reduces 5-HT synthesis [34].

This study showed that association between heat stress and intestinal infection or co-infection with *Eimeria* spp. and *C. perfringens* decreased the concentrations of NOR + AD in the hypothalamus and rostral pallium. In this sense, greater intensity and duration of stress reduced the concentration of catecholamines in the rostral pallium and in hypothalamus of birds. As suggested by Calefi et al. [27] and Vandenborne et al. [35], continuous production of corticosterone induced a high level of chronic stress in broilers.

Heat stress *per se* reduced NOR + AD levels and increased those of MHPG in the rostral pallium, midbrain, hypothalamus, and brainstem (suggesting increased catecholamine activity) and, at the same time, increased the dopamine turnover measured by the DOPAC/DA ratio in these same regions. Therefore, our experimental model produced, in fact, an intense chronic stress, observed in this study by the increase in central catecholaminergic activity as has been seen with other stressors applied to mammals [15]. In particular, these findings seems to show that the dopaminergic system also participates in the response to stressed birds, similar to the response found in mammals subjected to acute or chronic stress [36,37].

According to Sheridan [38], the release of NOR + AD in the hypothalamus produces activation of the HPA axis and consequent release of corticosterone. Corticosterone modulates the migration of heterophile to the site of infection and thus modulates the lesion intensity [21]. Calefi et al. [16] showed that infection with *C. perfringens* type A activated hypothalamic regions related to the HPA axis; further, they showed that an association between the infection and chronic heat stress increased the activity of hypothalamic neurons and triggered negative feedback, thus, leading to a reduction in serum corticosterone levels, as reported by Vandenborne et al. [35] in other conditions. These data agree with the profile of corticosterone and brain catecholaminergic activity found in animals of the C/HS34, Ei/HS34, Cp/HS34, and Ei + Cp/HS34 groups.

In addition to involving the catecholaminergic system, we found that heat stress *per se* influenced the serotonergic system. Indeed, heat stress decreased the levels of 5-HT and increased the turnover of 5-HT as observed through the 5-HIAA/5-HT ratio in the hypothalamus, midbrain, and brainstem of birds. These data showed an increase of serotonergic activity in these areas. Changes found in 5-HT concentrations and in 5-HT turnover agree with what has been reported in the hypothalamus and brainstem of animals subjected to chronic stress [13,14,15]. However, it is noteworthy that heat stress did not change the 5 -HT levels or the ratio of 5-HIAA/5-HT in the rostral pallium of birds.

The visualization of the neurochemical findings between the different brain structures analyzed in heat stressed animals makes it clear that the hypothalamus, midbrain, and brainstem have similar neurochemical profile changes. However, as already mentioned for 5-HT, small differences in the neurochemical profile were observed in the rostral pallium of the birds. This is probably because this brain structure has very different neural activities. The activity of the pallium has been associated with complex cognitive functions, while those of the hypothalamus, brainstem, and midbrain have been more related to the motor, endocrine, and autonomic functions, among others, and are also involved with the distribution of nerve stimuli to cortical regions [39].

The data from chronically heat stressed animals present a neurochemical profile consistent with the expression of sickness behavior, such as increase in 5-HT concentration observed in the rostral pallium, a decline in social interaction, the presence of depressive-like behavior, activation of the HPA axis, and a decrease in feed and water consumption [16,28,40,41,42]. In fact, Calefi et al. [16] observed that a chronic infection with *C. perfringens* produced behavioral changes characterized by a lower frequency of locomotion and feeding and an increase in expression of sleep behavior.

Future experiments relating the transcriptome profiles, behavioral changes, cerebral neurochemistry, and cytokine profile in the different biological systems can be used as a way to elucidate the changes observed in this work. In addition, in elucidating such pathways it is possible to explore pharmacological manipulation mechanisms to improve animal welfare and animal production.

## 5. Conclusions

As demonstrated, the more damaging the infectious process in the intestinal epithelium of birds is, the higher the activation of the CNS, a fact confirmed in this study by the modulation of brain monoamines, potentially due to the activation of the brain-gut axis. Furthermore, chronic heat stress and infection with *C. perfringens* and *Eimeria* spp. acted synergistically in the activation of neural pathways related to stress, and intestinal infections in birds are stressors *per se*. Finally, this study is relevant not only for understanding the pathophysiology of NE, but also for a better understanding of the neural pathways related to the gut-brain axis interactions in chickens.

## Supplementary Materials

The following are available online at http://www.mdpi.com/2306-7381/6/1/4/s1, Table S1. Quantification of neurotransmitters in the rostral pallium (average ± SE); Table S2. Quantification of neurotransmitters in the hypothalamus (average ± SE); Table S3. Quantification of neurotransmitters in the midbrain (average ± SE); Table S4. Quantification of neurotransmitters in the brainsteam (average ± SE).

## Abbreviations

The following abbreviations are used in this manuscript:

5-HIAA	5-hydroxyindoleacetic acid
5-HT	5-hydroxytryptamine
AD	epinephrine
BBB	blood-brain-barrier
CNS	central nervous system
DA	dopamine
DOPAC	4,4 dihydroxyphenylacetic acid
DHBA	3,4-dihydroxybenzylamine
ED	experimental day
HVA	homovanillic acid
HEPA	high-efficiency particulate air filters
HPA	hypothalamus-pituitary-adrenal
MHPG	3-methoxy-4-hydroxyphenylethylene glycol
NOR	Noradrenaline
POM	medial preoptic nucleus
PVN	paraventricular nucleus
VMA	vanillylmandelic acid
