# Supplementary material for: Heat Stress Modulates Brain Monoamines and Their Metabolites Production in Broiler Chickens Co-Infected with Clostridium perfringens Type A and Eimeria spp
Source: Vet Sci. 2019 Jan 9;6(1):4. doi: 10.3390/vetsci6010004 (PMC6466424; doi:10.3390/vetsci6010004)
Supplement: Supplementary file 1 [file vetsci-06-00004-s001.docx]

Supplementary Materials: Heat Stress Modulates Brain Monoamines and Their Metabolites Production in Broiler Chickens Co-Infected with Clostridium perfringens Type A and Eimeria spp.

Calefi A.S. *, Fonseca J.G.S., Nunes C.A.Q., Lima A.P.N., Quinteiro-Filho W.M. and Flório J.C.,

**Zager A., Ferreira A.J.P. and Palermo-Neto J**

Department of Pathology, School of Veterinary Medicine and Animal Science, University of São Paulo,

Av. Prof. Dr. Orlando Marques de Paiva, 87, São Paulo 05508-900, SP, Brazil; atilio@usp.br (C.A.S.);

julianagdsf@gmail.com (F.J.G.S.); catarina.queirooz@gmail.com (N.C.A.Q.); paulanlima@gmail.com (L.A.P.N.); quinteirofilho@gmail.com (Q.-F.W.M.); jflorio@usp.br (F.J.C.); adrianozager@gmail.com (Z.A.);

ajpferr@usp.br (F.A.J.P.); jpalermo@usp.br (P.-N.J.)

*****Correspondence: atilio@usp.br

**Table S1.** Quantification of neurotransmitters in the rostral pallium (average ± SE).

| **neurotransmitter** | **C** | **C/HS34** | **Ei** | **Ei/HS34** | **Cp** | **Cp/HS34** | **Ei + Cp** | **Ei + Cp/HS34** |
| --- | --- | --- | --- | --- | --- | --- | --- | --- |
| DA | 123 ^a^  ± 19 | 268 ^a^  ± 99 | 90 ^a^  ± 11 | 241 ^a^  ± 94 | 189 ^a^  ± 59 | 148 ^a^  ± 51 | 253 ^a^  ± 65 | 289 ^a^  ± 89 |
| DOPAC | 38 ^a^  ± 14 | 99 ^b^  ± 17 | 29 ^a^  ± 3 | 118 ^b,c^  ± 67 | 37 ^a^  ± 20 | 101^b^  ± 39 | 47 ^a,c^  ± 21 | 84 ^a,b^  ± 31 |
| HVA | 187 ^a^  ± 80 | 123 ^a^  ± 30 | 86 ^a^  ± 125 | 198 ^a^  ± 45 | 247 ^a^  ± 119 | 156 ^a^  ± 35 | 247 ^a^  ± 100 | 184 ^a^  ± 52 |
| 5-HT | 688 ^a^  ± 69 | 1041 ^b^  ± 60 | 757 ^a^  ± 80 | 1055 ^b^  ± 95 | 680 ^a^  ± 34 | 1064 ^b^  ± 65 | 771 ^a,b^  ± 65 | 932 ^a,b^  ± 78 |
| 5-HIAA | 95 ^a^  ± 14 | 142 ^a,b^  ± 12 | 167 ^a,b^  ± 10 | 158 ^a,b^  ± 18 | 95 ^a^  ± 15 | 191 ^b^  ± 18 | 158 ^a,b^  ± 44 | 144 ^a,b^  ± 20 |
| NOR+AD | 287 ^a^  ± 28 | 72 ^b^  ± 7 | 264 ^a^  ± 31 | 55 ^b^  ± 6 | 247 ^a^  ± 22 | 90 ^a,b^  ± 19 | 476 ^a^  ± 116 | 47 ^b^  ± 7 |
| MHPG | NA ^a^ | 148 ^b^  ± 13 | NA ^a^ | 176 ^b^  ± 10 | NA ^a^ | 172 ^b^  ± 15 | NA ^a^ | 151 ^b^  ± 15 |
| 5-HIAA/5-HT | 0.14 ^a^  ± 0.015 | 0.13 ^a^  ± 0.008 | 0.15 ^a^  ± 0.01 | 0.16 ^a^  ± 0.03 | 0.13 ^a^  ± 0.02 | 0.18 ^a^  ± 0.05 | 0.18 ^a^  ± 0.10 | 0.16 ^a^  ± 0.02 |
| DOPAC/DA | 0.21 ^a^  ± 0.03 | 0.6 ^a,b^  2 ± 0.18 | 0.36 ^a,b^  ± 0.04 | 0.71 ^a,b^  ± 0.51 | 0.27 ^a,b^  ± 0.23 | 1.31 ^b^  ± 0.23 | 0.38 ^a,b^  ± 0.15 | 0.70 ^a,b^  ± 0.21 |
| HVA/DA | 2.28 ^a^  ± 1.23 | 0.48 ^a^  ± 0.09 | 1.19 ^a^  ± 0.65 | 0.81 ^a^  ± 0.20 | 2.36 ^a^  ± 1.21 | 1.36 ^a^  ± 0.58 | 2.22 ^a^  ± 1.23 | 1.19 ^a^  ± 0.33 |

Note: values of DA (dopamine), DOPAC (4,4 dihydroxyphenylacetic acid), HVA (homovanillic acid), 5-HT (5-hydroxytryptamine), 5-HIAA (5-hydroxyindoleacetic acid), DA and NOR (Noradrenaline)+AD and MHPG (3-methoxy-4-hydroxyphenylethylene glycol) are expressed in ng/g of tissue. Different letters superscript on the averages indicate significant differences with *p* < 0.05 (Tukey HSD test). Control group (C); Group infected with *Eimeria* spp. (Ei); group infected with *Clostridium perfringens* (Cp); coinfected group with *Clostridium perfringens* and *Eimeria* spp. (Ei+Cp). The “/HS35” indicates the groups stressed by heat.

**Table S2.** Quantification of neurotransmitters in the hypothalamus (average ± SE).

| **neurotransmitter** | **C** | **C/HS34** | **Ei** | **Ei/HS34** | **Cp** | **Cp/HS34** | **Ei + Cp** | **Ei + Cp/HS34** |
| --- | --- | --- | --- | --- | --- | --- | --- | --- |
| DA | 554 ^a,b^  ± 112 | 406 ^a,b^  ± 60 | 441 ^a,b^  ± 93 | 309 ^a,b^  ± 26 | 580 ^b^  ± 88 | 320 ^a^  ± 45 | 610 ^b^  ± 44 | 252 ^a^  ± 36 |
| DOPAC | 104 ^a^  ± 12 | 159 ^a^  ± 20 | 96 ^a^  ± 19 | 123 ^a^  ± 8 | 111 ^a^  ± 12 | 140 ^a^  ± 13 | 114 ^a^  ± 11 | 98 ^a^  ± 18 |
| HVA | 144 ^a^  ± 27 | 1368 ^b^  ± 101 | 116 ^a^  ± 29 | 1522 ^b^  ± 239 | 159 ^a^  ± 80 | 1205 ^b^  ± 128 | 217 ^a^  ± 38 | 997 ^b^  ± 163 |
| 5-HT | 1845 ^a^  ± 203 | 864 ^b^  ± 95 | 1580 ^a^  ± 262 | 849 ^b^  ± 120 | 1596 ^a^  ± 213 | 825 ^b^  ± 126 | 2091 ^a^  ± 121 | 754 ^b^  ± 156 |
| 5-HIAA | 447 ^a^  ± 41 | 627 ^a^  ± 68 | 445 ^a^  ± 78 | 544 ^a^  ± 35 | 467 ^a^  ± 53 | 587 ^a^  ± 39 | 609 ^a^  ± 60 | 402 ^a^  ± 80 |
| NOR+AD | 1478 ^a^  ± 212 | 110 ^b^  ± 12 | 1467 ^a^  ± 219 | 105 ^b^  ± 21 | 1664 ^a^  ± 259 | 142 ^b^  ± 35 | 2289 ^a^  ± 108 | 114 ^b^  ± 24 |
| MHPG | NA ^a^ | 1443 ^b^  ± 95 | NA | 1330 ^b^  ± 127 | NA ^a^ | 1338 ^b^  ± 118 | NA ^a^ | 1171 ^b^  ± 174 |
| 5-HIAA/5-HT | 0.20 ^a^  ± 0.01 | 0.72 ^a,b^  ± 0.09 | 0.28 ^a^  ± 0.03 | 0.55 ^a,b^  ± 0.09 | 0.26 ^a^  ± 0.02 | 0.87 ^a,b^  ± 0.13 | 0.28 ^a^  ± 0.01 | 1.45 ^b^  ± 0.62 |
| DOPAC/DA | 0.17 ^a,b^  ± 0.03 | 0.51 ^a,b^  ± 0.18 | 0.19 ^a,b^  ± 0.05 | 0.40 ^a,b^  ± 0.08 | 0.30 ^a,b^  ± 0.12 | 0.45 ^a,b^  ± 0.06 | 0.17 ^a^  ± 0.01 | 0.72 ^b^  ± 0.18 |
| HVA/DA | 0.12 ^a^  ± 0.03 | 4.40 ^b^  ± 1.09 | 0.36 ^a^  ± 0.11 | 5.50 ^b^  ± 1.05 | 0.39 ^a^  ± 0.13 | 4.47 ^b^  ± 0.84 | 0.32 ^a^  ± 0.05 | 4.04 ^b^  ± 0.43 |

Note: values of DA (dopamine), DOPAC (4,4 dihydroxyphenylacetic acid), HVA (homovanillic acid), DA and NOR(Noradrenaline) + AD and MHPG (3-methoxy-4-hydroxyphenylethylene glycol) are expressed in ng/g of tissue. Different letters superscript on the averages indicate significant differences with *p* < 0.05 (Tukey HSD test). Control group (C); Group infected with *Eimeria* spp. (Ei); group infected with *Clostridium perfringens* (Cp); coinfected group with *Clostridium perfringens* and *Eimeria* spp. (Ei + Cp). The “/HS35” indicates the groups stressed by heat.

**Table S3.** Quantification of neurotransmitters in the midbrain (average ± SE).

| **neurotransmitter** | **C** | **C/HS34** | **Ei** | **Ei/HS34** | **Cp** | **Cp/HS34** | **Ei + Cp** | **Ei + Cp/HS34** |
| --- | --- | --- | --- | --- | --- | --- | --- | --- |
| DA | 406 ^a^  ± 43 | 191 ^b^  ± 68 | 401 ^a^  ± 55 | 105 ^b^  ± 19 | 480 ^a^  ± 52 | 115 ^b^  ± 20 | 338 ^a^  ± 71 | 113 ^b^  ± 27 |
| DOPAC | 78 ^a^  ± 12 | 94 ^a^  ± 11 | 96 ^a^  ± 18 | 73 ^a^  ± 21 | 128 ^a^  ± 25 | 63 ^a^  ± 9 | 67 ^a^  ± 13 | 78 ^a^  ± 19 |
| HVA | 229 ^a^  ± 68 | 144 ^a^  ± 35 | 214 ^a^  ± 75 | 187 ^a^  ± 69 | 342 ^a^  ± 128 | 152 ^a^  ± 39 | 132 ^a^  ± 19 | 235 ^a^  ± 61 |
| 5-HT | 1212 ^a^  ± 113 | 772 ^a,b^  ± 99 | 1398 ^a^  ± 103 | 627 ^b^  ± 93 | 1420 ^a^  ± 150 | 795 ^b^  ± 95 | 1329 ^a^  ± 154 | 712 ^b^  ± 111 |
| 5-HIAA | 346 ^a,b^  ± 34 | 363 ^a,b^  ± 41 | 512 ^a^  ± 76 | 271 ^b^  ± 38 | 396 ^a,b^  ± 47 | 375 ^a,b^  ± 61 | 402 ^a,b^  ± 35 | 251 ^a,b^  ± 39 |
| NOR+AD | 1168 ^a^  ± 119 | 126 ^b^  ± 24 | 1170 ^a^  ± 110 | 112 ^b^  ± 26 | 1276 ^a^  ± 201 | 97 ^b^  ± 29 | 1070 ^a^  ± 173 | 65 ^a^  ± 15 |
| MHPG | NA ^a^ | 605 ^b^  ± 66 | NA ^a^ | 593 ^b^  ± 65 | NA ^a^ | 466 ^b^  ± 115 | NA ^a^ | 641 ^a^  ± 101 |
| 5-HIAA/5-HT | 0.29 ^a^  ± 0.02 | 0.51 ^b^  ± 0.07 | 0.33 ^a,b^  ± 0.04 | 0.45 ^a,b^  ± 0.03 | 0.28 ^a^  ± 0.01 | 0.46 ^a,b^  ± 0.03 | 0.32 ^a,b^  ± 0.03 | 0.35 ^a,b^  ± 0.03 |
| DOPAC/DA | 0.19 ^a^  ± 0.02 | 0.78 ^a,b^  ± 0.15 | 0.21 ^b^  ± 0.10 | 0.86 ^a^  ± 0.08 | 0.32 ^a,b^  ± 0.02 | 0.59 ^a,b^  ± 0.26 | 0.23 ^s,b^  ± 0.02 | 0.87 ^a^  ± 0.25 |
| HVA/DA | 0.76 ^a^  ± 68 | 1.23 ^a^  ± 35 | 0.60 ^a^  ± 75 | 1.65 ^a^  ± 69 | 0.94 ^a^  ± 128 | 1.03 ^a^  ± 39 | 0.69 ^a^  ± 19 | 1.52 ^a^  ± 61 |

Note: values of of DA (dopamine), DOPAC (4,4 dihydroxyphenylacetic acid), HVA (homovanillic acid), DA and NOR(Noradrenaline) + AD and MHPG (3-methoxy-4-hydroxyphenylethylene glycol) are expressed in ng/g of tissue. Different letters superscript on the averages indicate significant differences with *p* < 0.05 (Tukey HSD test). Control group (C); Group infected with *Eimeria* spp. (Ei); group infected with *Clostridium perfringens* (Cp); coinfected group with *Clostridium perfringens* and *Eimeria* spp. (Ei + Cp). The “/HS35” indicates the groups stressed by heat.

**Table S4.** Quantification of neurotransmitters in the brainsteam (average ± SE).

| **neurotransmitter** | **C** | **C/HS34** | **Ei** | **Ei/HS34** | **Cp** | **Cp/HS34** | **Ei + Cp** | **Ei + Cp/HS34** |
| --- | --- | --- | --- | --- | --- | --- | --- | --- |
| DA | 232 ^a^  ± 18 | 199 ^a^  ± 27 | 185 ^a^  ± 14 | 168 ^a^  ± 13 | 178 ^a^  ± 6 | 183 ^a^  ± 28 | 178 ^a^  ± 18 | 180 ^a^  ± 18 |
| DOPAC | 44 ^a^  ± 13 | 207 ^b^  ± 22 | 23 ^a^  ± 3 | 188 ^b^  ± 23 | 35 ^a^  ± 6 | 215 ^a^  ± 20 | 51 ^b^  ± 10 | 222 ^a^  ± 17 |
| HVA | 52 ^a^  ± 4 | 247 ^a^  ± 93 | 63 ^a^  ± 11 | 128 ^a^  ± 42 | 110 ^a^  ± 62 | 201 ^a^  ± 69 | 65 ^a^  ± 13 | 235 ^a^  ± 64 |
| 5-HT | 1298 ^a^  ± 136 | 1073 ^a^  ± 170 | 1370 ^a^  ± 148 | 1117 ^a^  ± 62 | 1353 ^a^  ± 46 | 1081 ^a^  ± 95 | 1358 ^a^  ± 115 | 908 ^a^  ± 98 |
| 5-HIAA | 338 ^a^  ± 32 | 467 ^a^  ± 36 | 432 ^a^  ± 25 | 402 ^a^  ± 30 | 353 ^a^  ± 30 | 442 ^a^  ± 47 | 355 ^a^  ± 32 | 329 ^a^  ± 49 |
| NOR+AD | 1111 ^a^  ± 64 | 201 ^b^  ± 40 | 1057 ^a^  ± 98 | 183 ^b^  ± 37 | 1070 ^a^  ± 70 | 222 ^b^  ± 19 | 1020 ^a^  ± 107 | 174 ^a^  ± 18 |
| MHPG | NA ^a^ | 1055 ^b^  ± 69 | NA ^a^ | 907 ^b^  ± 62 | NA ^a^ | 871 ^b^  ± 100 | NA ^a^ | 915 ^a^  ± 87 |
| 5-HIAA/5-HT | 0.28 ^a^  ± 0.02 | 0.71 ^b^  ± 0.21 | 0.31 ^a,b^  ± 0.03 | 0.36 ^a,b^  ± 0.01 | 0.27 ^a^  ± 0.03 | 0.40 ^a,b^  ± 0.03 | 0.30 ^a^  ± 0.03 | 0.42 ^a,b^  ± 0.11 |
| DOPAC/DA | 0.17 ^a^  ± 0.03 | 1.21 ^b^  ± 0.17 | 0.11 ^a^  ± 0.00 | 1.21 ^b^  ± 0.12 | 0.19 ^a^  ± 0.02 | 1.27 ^b^  ± 0.19 | 0.28 ^a^  ± 0.05 | 1.29 ^b^  ± 0.10 |
| HVA/DA | 0.24 ^a^  ± 0.02 | 1.69 ^a^  ± 0.88 | 0.29 ^a^  ± 0.02 | 0.85 ^a^  ± 0.30 | 0.58 ^a^  ± 0.29 | 1.45 ^a^  ± 0.62 | 0.36 ^a^  ± 0.07 | 1.34 ^a^  ± 0.38 |

Note: values of of DA (dopamine), DOPAC (4,4 dihydroxyphenylacetic acid), HVA (homovanillic acid), DA and NOR(Noradrenaline) + AD and MHPG (3-methoxy-4-hydroxyphenylethylene glycol) are expressed in ng/g of tissue. Different letters superscript on the averages indicate significant differences with *p* < 0.05 (Tukey HSD test). Control group (C); Group infected with *Eimeria* spp. (Ei); group infected with *Clostridium perfringens* (Cp); coinfected group with *Clostridium perfringens* and *Eimeria* spp. (Ei + Cp). The “/HS35” indicates the groups stressed by heat.
